# Environment-Friendly Preparation of Reaction-Bonded Silicon Carbide by Addition of Boron in the Silicon Melt

**DOI:** 10.3390/ma14051090

**Published:** 2021-02-26

**Authors:** Maoqiang Rui, Yaxiang Zhang, Jing Ye

**Affiliations:** School of Material Science and Engineering, Wuhan University of Technology, Wuhan 430070, China; rmq15623897961@whut.edu.cn (M.R.); zhangyx@whut.edu.cn (Y.Z.)

**Keywords:** reaction-bonded silicon carbide, B–Si alloy, infiltration

## Abstract

Reaction-bonded silicon carbide ceramics were sintered by infiltration of Si and B–Si alloy under an argon atmosphere at different temperatures. The element boron was added to the silicon melt to form a B–Si alloy first. The mechanical properties of samples were improved by infiltration of the B–Si melt. The samples infiltrated with the Si-only melt were found to be very sensitive to experimental temperature. The bending strengths of 58.6 and 317.0 MPa were achieved at 1530 and 1570 °C, respectively. The sample made by infiltration of B–Si alloy was successfully sintered at 1530 °C. The relative density of the sample was more than 90%. The infiltration of B–Si alloy reduced the sintering temperature and the bending strength reached 326.9 MPa. The infiltration mechanism of B–Si alloy is discussed herein.

## 1. Introduction

SiC ceramics are always applied as high temperature structural components due to the advantages of their mechanical properties at high temperature. The SiC rollers, which are usually used in tunnel kilns, are one of the most important materials for the production of architectural and sanitary ceramics. Re-crystallization of SiC and reaction bonding of silicon carbide (reaction-bonded silicon carbide—RBSC) are the main methods for the manufacturing of near-net shaped SiC. The re-crystallization method demands a sintering temperature of more than 2200 °C, which consumes lots of energy. By contrast, reaction-bonding silicon carbide has been found to be an environment-friendly way to produce SiC because of its lower temperature preparation [1,2,3,4]. The main mechanism of reaction-bonding silicon carbide is as follows: at a temperature beyond the melting point of silicon, the silicon melt penetrates into the green body through capillary force, reacts with residual carbon in the SiC green body and forms β-SiC. However, free silicon in the ceramics makes the mechanical properties of SiC ceramics poor [5]. The RBSC is always disposed of as solid waste after utilization at a high temperature. Consequently, many methods are applied to reduce the free-silicon content in ceramics.

The physical properties of RBSC have been constantly advanced. The bending strength of RBSC increased from 300 to 600 MPa [6,7,8], the hardness of RBSC has been reported to be 25 GPa and its relative density reached 3.15 g/cm^3^ [9,10]. On the other hand, most studies are inclined to produce RBSC via vacuum sintering according to extensive experimental verification. The experimental results from the literature showed that even at a high sintering temperature or with a long holding time [11], the bending strength of RBSC sintered under an Ar atmosphere was not nearly as optimal as RBSC produced through vacuum sintering. In fact, vacuum sintering demands stringent specifications of the equipment. The output of RBSC is limited by the production conditions, which causes higher costs consequently. However, pressureless sintering is a comparatively simple method that could scale up the production and lower the cost.

Thus, the present research focuses on an environment-friendly version of RBSC sintering. Boron is added into Si melt in order to reduce the sintering temperature of the specimen under an argon atmosphere and improve the mechanical properties. The mechanisms of the penetration of different melt are also discussed in present study.

## 2. Materials and Methods

A sample RBSC ceramic was fabricated under an Ar atmosphere in the present study to investigate the effect of the addition of boron into the silicon melt on the ceramic sintering. To confirm the smooth infiltration of different melts into the green body, the B–Si alloy of eutectic point was prepared before the experiment. The chemicals used in RBSC ceramic preparation along with their purity levels and suppliers are listed in Table 1.

### 2.1. B–Si Alloy Preparation

The B–Si alloy was prepared first by mixing B and Si powders. According to the phase diagram [12], the lowest eutectic temperature of B–Si is 1385 °C at the silicon-rich side with a silicon content of 97 wt.% (92 at.%) in the binary system. The B and Si powders in desired proportions were wet-mixed by using ethanol as the ball-milling medium in a planetary ball mill (XGB2, produced by Nanjing Boyuntong Instrument Technology Co., Ltd., Nanjing, China) for 4 h (Agate ball mill beads). After drying, the mixture was placed in s tube furnace and melt at 1500 °C in a BN crucible under a pure Ar atmosphere. The alloy was taken out from furnace after cooling and crushed into small pieces of 1–3 mm.

### 2.2. Materials and Sample Preparation

The batch formulas of different samples are listed in Table 2. The utilization of phenolic resin could enhance the strength of ceramic green body and also provided the carbon for the RBSC ceramic. The phenolic resin used in this study contained 35 wt.% of carbon after pyrolysis. Thus, the carbon in the ceramic green body was from two sources, carbon black and phenolic resin.

According to the batch formula, the raw materials were thoroughly wet-mixed in a planetary ball mill (XGB2, produced by Nanjing Boyuntong Instrument Technology Co., Ltd., Nanjing, China) for 4 h with rotation speed of 200 r/min by using ethanol as the ball-milling medium (Agate ball mill beads). The mixed powders were dried at 80 °C for 24 h in an oven. To improve the plasticity of mixture, the powders after drying were pelleted by adding 7 wt.% polyvinyl alcohol (PVA) aqueous (with a concentration of 5 wt.%), and aged for 24 h. The mixtures were then pressed unidirectionally into rectangular bars (37 mm × 6 mm × 3 mm) under a pressure of 120 MPa. The green bodies were dried at 80 °C for 24 h and placed in alumina crucible. To avoid the influences of heat release and gas generation during RBSC processing, the binder removal process was taken first. The green bodies were placed in the tube furnace and heated slowly to 800 °C at a heating rate of 0.5 °C/min under an Ar atmosphere.

**Figure 1:**
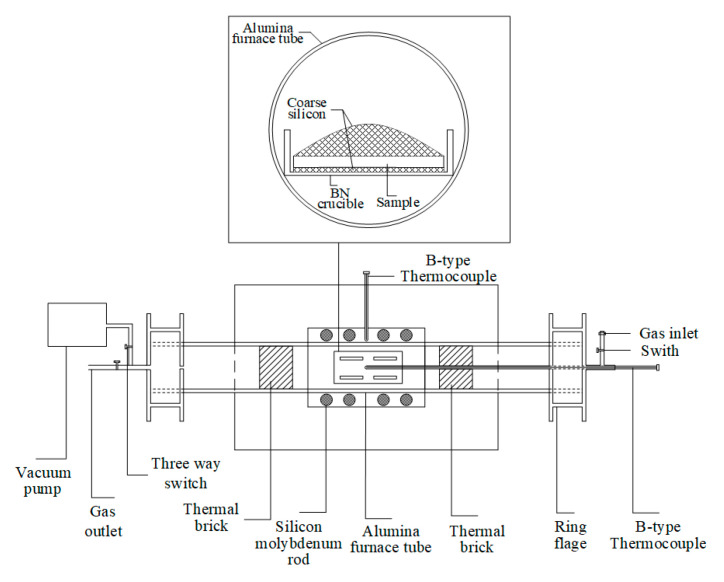
Experimental setup.

After that, the green bodies were carefully placed in the BN crucible and covered by Si or B–Si alloy pieces. A high-temperature tube furnace with MoSi_2_ heating elements was employed, which enabled a maximum temperature of 1700 °C. The experimental setup is presented in Figure 1. The horizontal alumina tube was a reaction chamber, which was sealed gas tight with O-rings at both ends by a stainless-steel flange arrangement. A B-type thermocouple was introduced in the reaction chamber to read the real temperature of the samples. The samples along with the BN crucible were covered with a lid and placed just below the B-type thermocouple in the reaction chamber. The reaction chamber was evacuated and then back-filled with Ar gas. To ensure that most of the air was flushed away, this procedure was repeated three times before the furnace was heated up. Finally, the tube was filled with Ar gas, and an argon flow of about 0.1 L/min was maintained throughout the whole experiment. Subsequently, the furnace was heated up to a predetermined temperature with a heating rate of 3 °C/min to protect the alumina tube from thermal shock and hold it at a predetermined temperature for 3 h.

### 2.3. Sample Analysis

The phase of the B–Si alloy after preparation was identified by X-ray diffraction (XRD). A D/MAX-IIIA diffractometer (Rigaku Corporation, Tokyo, Japan) equipped with Cu Kα (λ = 1.54 Å) radiation was employed for this analysis. The scanning angle (2θ) was tested from 5° to 80°with step of 0.02°. A scanning electron microscope (QUANTA FEG 450, FEI, USA) was introduced to analyze the microstructures of different samples. Each bulk density was tested via Archimedes’ method to calculate the relative densities of different samples. The bending strengths of different samples after the experiment were evaluated through a three-point bending mode by using a computer-controlled electronic universal testing machine (QJ211S-10KN, produced by Shanghai Qingji Testing Instruments CO., LT, Shanghai, China). The span for the testing was 30 mm and the test-machine crosshead displacement rate was set at 0.5 mm/min, and at least five specimens were measured for each group of samples. The HVT-1000 micro hardness tester was employed to measure the Vickers hardness of the samples. The surfaces of the samples were polished before the test, the load was set at 9.8 N and held for 10 s; reported values were averaged from at least five measurements.

## 3. Results

**Figure 2:**
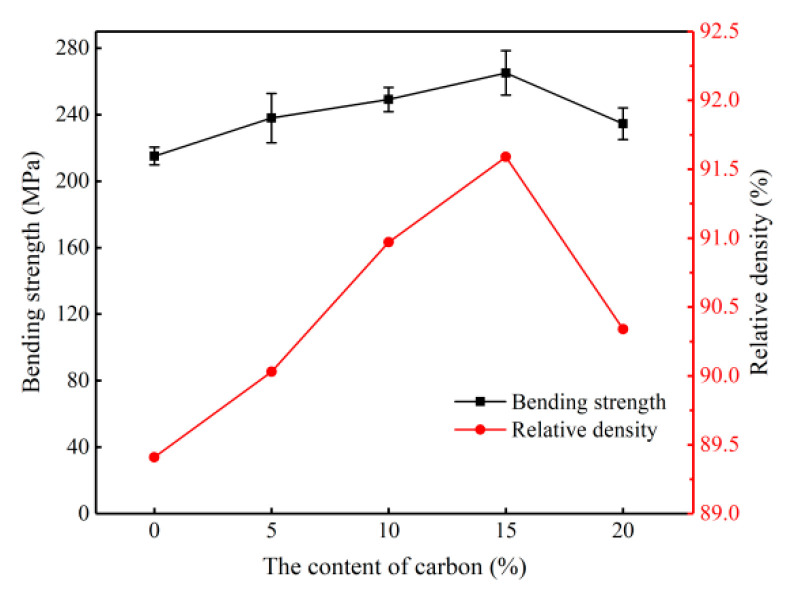
Relative density and bending strength values of siliconized sintered samples at 1600 °C under an Ar atmosphere.

To determine the best batch formula among the different samples, the penetration of Si melt into green bodies was carried out first at 1600 °C under an argon atmosphere. The relations between additional content of carbon black and comprehensive performances of different samples are presented in Figure 2. Considerable improvements in relative density (91.6%) and bending strength (265.1 MPa) were gained with the addition of 15 wt.% of carbon black. However, the addition of beyond 15 wt.% of carbon black causes strong decreases in relative density and bending strength. Consequently, sample S4 with the addition of 15 wt.% carbon black was used for the experiments of Si and B–Si penetration based on the experimental results.

**Figure 3:**
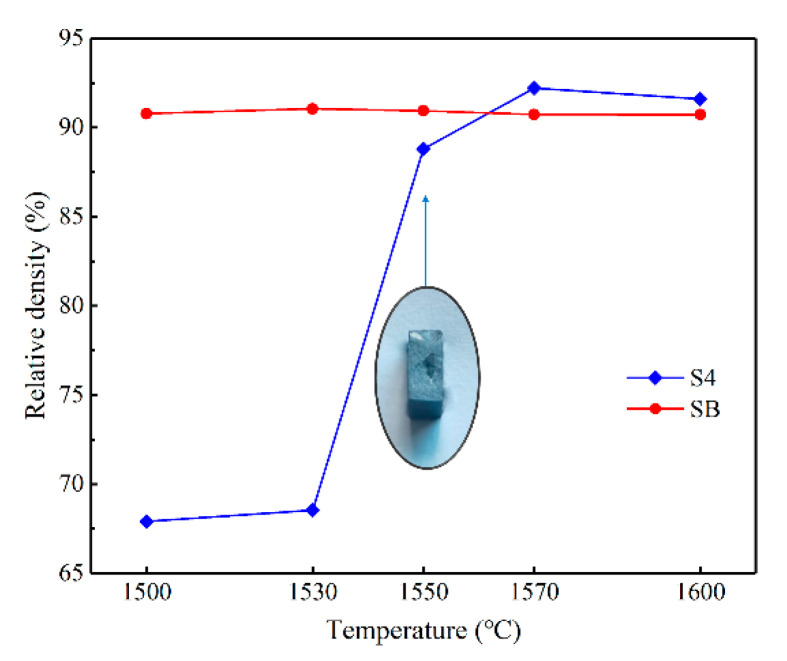
Relative densities of samples S4 and SB as functions of firing temperature.

To understand the densification process of RBSC ceramics during heating, the variation of density as function of reaction temperature is shown in Figure 3. A convincing show of density change was observed by infiltration of molten silicon. Sample S4 with a relative density of 68.5% was obtained at an infiltration temperature of 1530 °C, which means that very limited Si melt infiltrated into the green body. However, the relative densities of 88.8% and 92.2% were achieved at infiltration temperatures of 1550 and 1570 °C, respectively. Sample S4 after reaction at 1550 °C was cut transversally. Ocular examination revealed that even at 1550 °C, the Si melt did not fully infiltrate into the ceramic and an unreacted region was clearly observed. However, differing from Si infiltration, a slight variation of density occurred when the reaction temperature rose with infiltration of molten B–Si alloy. The relative density of about 90% was achieved at different temperatures, as shown in Figure 3. The density of samples infiltrated by molten B–Si alloy was observed to be slightly lower than that of the sample with highest density by infiltration of molten Si melt.

**Figure 4:**
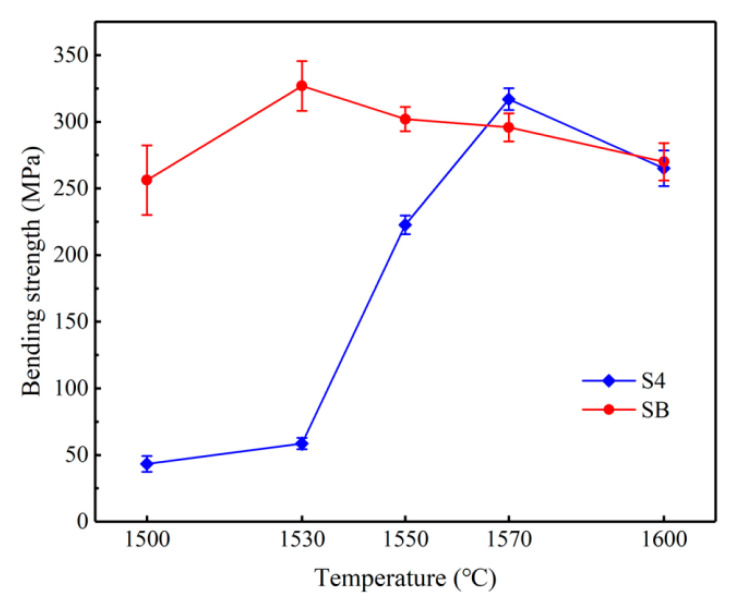
Bending strengths of samples S4 and SB as functions of firing temperature.

Figure 4 presents the bending strengths of different types of samples after experiments as functions of infiltration temperature. The bending strength is defined as:

(1) $$\sigma_{b}=\frac{3\mathrm{FL}}{2{\mathrm{BH}}^{2}}$$

where σ_b_ is the bending strength (MPa), F is the maximum load at fracture (N), L is the span (mm), B is the sample width (mm) and H is the sample height (mm). The results reflect continued improvements in bending strength of sample S4 with rises in temperature. The bending strengths of 58.6 and 317.0 MPa were achieved at 1530 and 1570 °C. Differently from sample S4, sample SB showed that the highest bending strength of 326.9 MPa was obtained at temperature of 1530 °C. The bending strength of the sample continuously fell at temperatures beyond 1530 °C, but the maximum decrease in bending strength was no more than 50 MPa.

**Figure 5:**
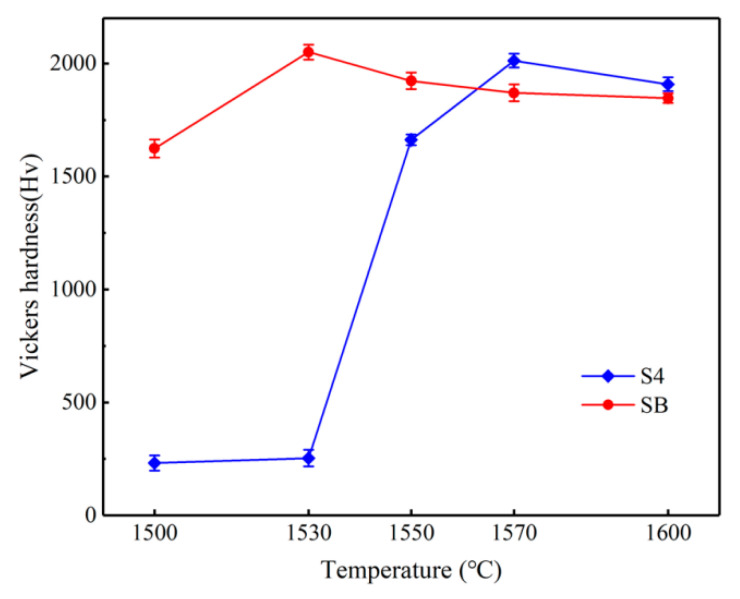
Vickers hardness of samples S4 and SB as a function of firing temperature.

Figure 5 shows the relation of sample hardness and temperature. Compared with Figure 4, similar trends of these two types of samples are presented in Figure 5. The hardness of a sample is strongly related to the experimental temperature. As the temperature rose, the hardness of the sample improved accordingly. The hardness of 2000 Hv was achieved at the experimental temperature of 1570 °C, which is a maximum increase of 1600 Hv in the hardness of sample. Additionally, a slightly decrease in hardness showed up beyond 1570 °C. On the other hand, the experimental temperature of 1530 °C made the sample hardest. It is noteworthy that the samples with B–Si melt infiltration at the best experimental temperature exhibited better mechanical properties, as shown in Figure 4 and Figure 5. Thus, the microstructures of the samples were examined by SEM.

**Figure 6:**
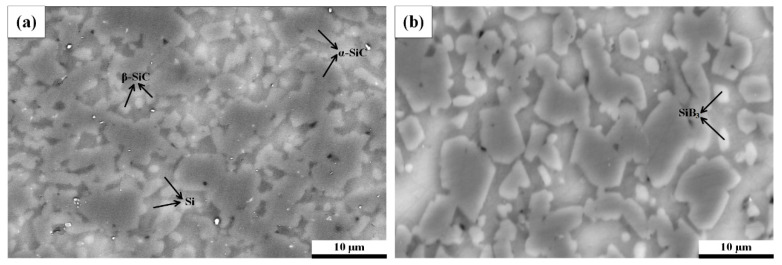
Backscatter electron images (BSEI) of samples S4 and SB, which were sintered at different temperatures under an Ar atmosphere: (**a**) sample S4; (**b**) sample SB.

**Figure 7:**
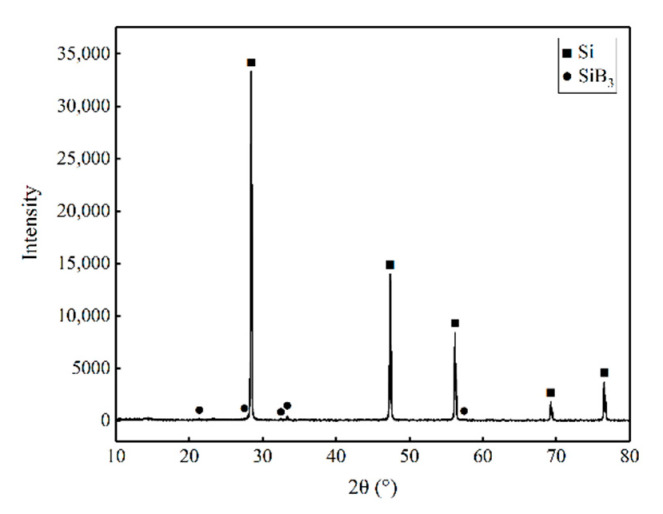
XRD patterns of B–Si alloy sintered at 1500 °C under an Ar atmosphere.

Figure 6a,b presents the SEM micrographs of S4 and SB after experimentation at 1570 and 1530 °C, respectively. Figure 6a shows sample S4 after infiltration of Si melt at 1570 °C with ×2500 magnification. Three different phases are identifiable. It is reasonable to conclude that the dark, big phases are α-SiC in the green body and the small phases are β-SiC formed by Si melt infiltration and carbon black in the green body. The rest is Si. Sample SB after infiltration of B–Si alloy at 1530 °C with same magnification is presented in Figure 6b. Besides the three phases identified in Figure 6a, some small bright white phases are present in Figure 6b. Boron is also a light element which is not suitable for EDS analysis. However, as shown in Figure 7, according to the XRD analysis, those small pieces are SiB_3_.

## 4. Discussion

### 4.1. The Influence of Carbon Content

As shown in Figure 2, sample S4 with the additional content of 15 wt.% carbon black showed the best comprehensive performance. The additional carbon in the SiC green body provides the opportunity of SiC formation. After silicon melting, the silicon melt infiltrates into the connected pores and reacts with the carbon in the green body. Consequently, SiC is formed according to the following reaction steps:

(2) Si(l)+C(graph)→Si/C(soln)

(3) Si/C(soln)→SiC(s)

The formation of SiC during the process enhances the mechanical properties and improves the relative density of SiC ceramics. The C (graph) appearing in Equation (2) easily produces molar volume expansion in the vertical direction between the layers during the reaction-bonding process due to its layered crystal structure, which eventually leads to the cracking of the sintered body. Therefore, this experiment used carbon black instead of graphite. On the other hand, as shown in Figure 6, carbon black can react with the infiltrated silicon melt, but it cannot eliminate the residential silicon; thus, the RBSC ceramics always contain free silicon. Even for sample S1, absent any carbon black, the RBSC ceramic was made dense by infiltration of the Si melt. The poor mechanical properties and low relative density of the sample were contributed by Si in consequence. The carbon black in the green body will make the RBSC ceramics denser. The explanation is given as follows [13]: when the carbon meets the Si melt, it dissolves into the melt, and after the melt has saturated, the β-SiC precipitates at energy-dense sites of α-SiC. Similar results are presented in Figure 6a.

Although more carbon black in the green body makes the RBSC ceramics have better physical properties, the additional amount still has its limits. The research shows that residential carbon particles in the ceramics which are introduced by too much carbon addition will cause undesirable consequences. Carbon black addition of 15 wt.% was found to be with the best amount in this study.

### 4.2. The Influence of Sintering Temperature

The kernel of RBSC ceramic sintering is the infiltration of the Si melt into the RBSC green body, because the reaction happens only after the infiltration. As shown in Figure 3 and Figure 4, the relative density of RBSC ceramics and the mechanical properties were very undesirable after experimentation at 1530 °C, and the penetration happened only at the surface level. A sharp rise in the physical properties was achieved after sintering at 1550 °C, but an ocular examination still revealed an unreacted core in the sample, which indicates that the sample was without full infiltration.

Silicon completely melts at above 1420 °C and the viscosity value of liquid silicon is 0.5820 mPa·s [14,15]. The infiltration of the Si melt into pores is mainly due to the capillary force. Thus, the silicon melt at 1530 °C should infiltrate into the pores if the capillary force is enough. Considering that the connected pores are small tubes, the penetration depth can be calculated by the following equation [16,17]:

(4) $$D=\frac{2\gamma \cos\theta }{\rho \mathrm{gr}}$$

where γ is the liquid–vapor surface energy, θ is the contact angle, ρ is the density of the silicon melt, g is gravity and r is the tube radius. The connected pores were created by SiC powders in the present study, and the contact angle between Si drops and SiC has been reported as 37° at 1430 °C [18], which implies that SiC tubes must be wetting. Thus, differing from the results, the silicon melt must infiltrate into the pores at 1530 °C.

On the other hand, the SiC green body is a mixture of carbon black and SiC powders; thus, the surface of the channel is covered by carbon, not only SiC. θ should be the contact angle of silicon and carbon. The contact angles of Si drops and carbon are reported to be 50° under vacuum and 120° under an Ar atmosphere, respectively, at 1430 °C [19,20]. Please note that the contact angle mentioned here is at the initial stage. which means the silicon just drops on the substrate, since later on, the silicon reacts with carbon and a SiC layer is formed, which makes the contact angle decrease continuously. The bigger difference between the contact angle under vacuum and an Ar atmosphere explains why a vacuum is most often chosen as the sintering atmosphere for producing RBSC ceramics. The initial contact angle θ^0^ can be deduced by the following equations [21]:

(5) $${\cos\theta }^{0}=\left(\;\frac{W_{e}}{\sigma_{\mathrm{lv}}}\;\right)-1$$

(6) $$W_{e}\propto \exp\left(-\frac{∆G}{\mathrm{RT}}\right)\cdot E_{k}$$

where θ^0^ is initial contact angle; W_e_ is the equilibrium contribution of the work adhesion; σ_lv_ is the surface tension of the melt; ΔG is reaction energy barrier; R is ideal gas constant; T is temperature in Kelvin; E_k_ is the bond energy of two atoms. Equations (5) and (6) reveal that the contact angle will decrease with an increase in temperature.

The results show that the infiltration of Si melt is limited, so the contact angle is calculated accordingly. The contact angles at 1530 and 1550 °C were calculated as around 81° and 71°, which indicates that the infiltration depth of the Si melt must have been limited, and the calculation results agree fairly well with the experimental results. Consequently, the β–SiC formed only on the surface of the sample and unexpected closing of connected pores in green bodies caused the retardation of Si penetration.

### 4.3. The Influence of B–Si Alloy

The results from Figure 3 show that sample SB using B–Si alloy as its infiltration melt had stable relative density at temperatures from 1500 to 1600 °C. The alloy reduces the contact angle between the melts due to a lower melting point and lower viscosity, which makes the B–Si melt completely infiltrate into the ceramics at 1500 °C.

The B–Si alloy infiltrated into the ceramic successfully, whereas the mechanical properties were found to be decreased. The reasons are concluded to be as follows:

To ensure the complete melting of the alloy at experimental temperature, the composition of the B–Si was set to the eutectic point at the silicon content of 97 wt.%. After the infiltration, the furnace started to cool. Due to the low viscosity of silicon melt, the liquid phase very easily precipitated Si and SiB_3_ phases according to the phase diagram. Figure 6b and Figure 7 prove the precipitation of Si and SiB_3_ phases during cooling. The mechanical properties are strongly related to free-silicon content; the formation of SiB_3_ reduced the free-silicon content and improved the mechanical properties.

## 5. Conclusions

The sintering of RBSC ceramics by infiltration of different melts under Ar atmosphere at different temperatures was studied. The physical properties of samples infiltrated with a Si melt were strongly related to the experimental temperature. The silicon melt was very poor at infiltrating into the pores of the RBSC green bodies at 1530 °C. Only 68.5% relative density was obtained. With the temperature rising, the infiltration condition was improved and 92.2% relative density was achieved. In this approach, SiC powders were covered by carbon; the contact angle of the Si melt and carbon was large. Temperature rising could reduce the contact angle and made the infiltration process a success.

On the other hand, the B–Si alloy melt completely infiltrated into the RBSC green bodies at 1500 °C. The small contact angle resulted from the work adhesion, which was enhanced by the B–Si alloy. The relative density was very stable with experimental temperatures from 1500 to 1600 °C, and the best mechanical properties were gained at 1530 °C.

## Figures and Tables

**Table 1:** Experimental reagents.

Materials	Purity	Manufacturer
α-SiC powder	AR	Kete New Material Technology Co., Ltd., Hangzhou, China
Carbon black	AR	Graphene Gold Mall Co., Ltd., Changzhou, China
Phenolic resin	CP	Borun New Material Technology Co., Ltd., Ningbo, China
Polyvinyl alcohol	AR	Sinopharm Chemical Reagent Co., Ltd., Shanghai, China
Silicon powder	AR	Shanghai Macklin Biochemical Co., Ltd., Shanghai, China
Boron powder	AR	Shanghai Macklin Biochemical Co., Ltd., Shanghai, China

**Table 2:** Batch formulas of different samples (wt.%).

Sample	α-SiC	Carbon Black	Phenolic Resin (Addition)
* S1	100	-	10
S2	95	5	10
S3	90	10	10
S4	85	15	10
S5	80	20	10
^#^ SB	85	15	10

Notation: * Series S represents infiltrated silicon melt. ^#^ Series SB represents infiltrated B–Si alloy.

## Data Availability

The data presented in this study are available on request from the corresponding author.
